# Memoir of Dr. George Sydney Jones

**Published:** 1875-08

**Authors:** W. H. Morgan


### ARTICLE II.

### *Memoir of Dr. George Sidney Jones.*

BY W. H. MORGAN, M. D., D. D. S.

Dr. George Sidney Jones was born in Cumberland County, Va May 27th, 1808, was reared in Buckingham County, Va., learned the cabinet making business in Lynchburg. In 1835, he moved to Addairville, Logan County, Ky., where he opened a cabinet maker's shop.

In 1847, he commenced the study of Dentistry, and in 1850 closed his shop, and from this forward he gave his entire time and energies to his new calling. He graduated from the Baltimore College of Dental Surgery in 1851, and soon thereafter located in Russellville, Ky., where he continued to practice with great success until the beginning of his last illness, which terminated in death April 25th, 1875. Dr. Jones was one of nature's nobleman, warm hearted, generous and frank, his ear was ever open to the poor, helpless or distressed, and his hand ready to supply their wants so far as his ability would permit. No man in his sphere and with like pecuniary ability, within the knowledge of the writer did more to gladden the widow's heart or dry the orphan's tears than he. He was especially the friend of young men, taking great pleasure in instructing and advising them, encouraging them by his oft repeated motto—" Qualify yourselves for usefulness and the world will use you." He belonged to the Mystic Order of F. & A. Masons, was given to charity and the practice of every virtue.

As a dentist, Dr. J. was eminently conservative, safe and skillful. No man in the professson had a higher appreciation of its dignity or sought more earnestly to promote its interests than he. He labored to elevate it in the estimation of his fellow men. He was present at the organization of the American Dental Association in 1860, and was the ardent friend of dental associations and dental colleges always frowning upon everything that smacked of quackery.

In 1850, he joined the Reformed or Christian Church, and from that evinced his love for his Heavenly Father by an upright walk and godly conversation, on all suitable occasions recommending and enforcing the truths of the Holy Bible as man's only hope for eternal salvation.

His last hours were calm and peaceful. With christian resignation he said, " not my will, but Thine be done." Such a triumph in death is only vouched safe to the Christian. " Let me die the death of the righteous, and let my last end be like His."

In Dr. J's death the dental profession has lost one who dignified his calling; the Masonic fraternity one who squared his life by the principles of the order; and the church a zealous member.

---

